# Gender differences in spousal caregiver strain and paid service use among dementia caregivers in rural Appalachia

**DOI:** 10.3389/fpubh.2025.1620744

**Published:** 2025-08-13

**Authors:** Jyoti Savla, Karen A. Roberto, Leslie A. Fontaine

**Affiliations:** ^1^Center for Gerontology, Virginia Tech, Blacksburg, VA, United States; ^2^Department of Human Development and Family Science, Virginia Tech, Blacksburg, VA, United States; ^3^Institute for Society, Culture and Environment, Virginia Tech, Blacksburg, VA, United States

**Keywords:** caregiver burden, loneliness, in-home care services, help-seeking behavior, gender norms, Stress Process Model, informal support, activities of daily living

## Abstract

Spousal caregivers of persons living with dementia (PLwD) often experience high overload and loneliness due to the intensive and ongoing nature of caregiving for their partner. Paid in-home services, such as assistance with daily household tasks, respite care, or personal care, might help ease caregivers' physical strain; however, it is unclear if these in-home services effectively address loneliness and overload and whether their benefits differ by gender. Guided by the Stress Process Model, we analyzed structured interview data from 61 spousal caregivers living in rural Appalachia. Although husbands reported significantly lower loneliness and overload than wives, the overall use of paid services was similar across husbands and wives. Among those experiencing high stress, however, a higher percentage of husbands used in-home paid services than wives. Logistic regression analyses revealed that greater emotional strain (overload and loneliness) and higher functional impairment of the PLwD were independently associated with increased likelihood of paid service use. Our findings underscore the need for programs and policies to acknowledge emotional strain as a legitimate criterion for eligibility for paid services.

## 1 Introduction

Family caregivers play a critical role in supporting the quality of life of persons living with dementia (PLwD), often preventing or delaying placement in assisted living facilities or nursing homes. Among these caregivers, spouses often assume the most sustained and intensive responsibilities, placing them at heightened risk for emotional and physical burden (1, 2). The daily demands of caregiving often constrain spousal caregivers' ability to leave their home or pursue self-care, especially when their partner cannot be left alone. These constraints, compounded by physical fatigue from hands-on care and managing household tasks and emotional exhaustion from managing dementia-related behavioral symptoms, can take a cumulative toll on caregivers' physical, emotional, and social wellbeing (3).

Two forms of emotional strain are especially prevalent among spousal caregivers: caregiver overload and loneliness. Prior research has consistently documented high levels of caregiver overload marked by constant vigilance, fatigue, and limited opportunities for personal time (4). Loneliness is defined as the subjective distress that arises when one's social relationships are perceived as deficient in quality and quantity. Contemporary researchers categorize loneliness into three interconnected facets: emotional loneliness (lack of close attachments), social loneliness (absence of a supportive network of friends and family), and existential loneliness (a feeling of meaninglessness) (5). For spousal caregivers, loneliness may manifest as feeling isolated, cut off from others, and a diminished sense of belonging. Both overload and loneliness are linked to poor mental and physical health, which in turn may compromise spouse caregiver's ability to provide care (6).

To cope with these challenges, some spousal caregivers turn to paid in-home services such as assistance with activities of daily living (ADLs), homemaking, personal care, respite, and rehabilitative therapies. These in-home care services can provide meaningful relief to caregivers by easing the physical and emotional demands of caregiving and creating opportunities for rest and social engagement with others. However, many caregivers, especially in rural regions, avoid or delay using paid care due to financial barriers, lack of awareness of available services, unavailability of services in their area, or discomfort with allowing outsiders into the home (7).

While paid in-home services may help relieve caregiving stress, their potential to alleviate emotional strain, particularly caregiver loneliness, remains less understood. Most research examines how paid services affect older adults with functional limitations, not caregivers specifically. For example, Arsenijevic and Groot, using SHARE data from nine European countries, found no association between reduced government-supported household help and increased loneliness among older adults (8). Conversely, drawing on the China Health and Retirement Longitudinal Study (CHARLS), researchers reported that using home- and community-based services was associated with lower loneliness among Chinese older adults with physical limitations (9). Whether in-home services confer similar emotional benefits for spousal caregivers of PLwD remains an open question.

In addition to emotional strain, caregivers' decision to seek support are shaped by gendered norms (10). While both men and women spousal caregivers of PLwD report elevated levels of caregiving strain, wives were more likely to report poorer mental health outcomes, including higher levels of depression and social isolation (11). These differences reflect not only personal risk factors but also broader social expectations, structural norms, and constraints. Women are more likely to be the primary caregivers, provide more hours of care, and receive less help from others (12). Connidis and McMullin's concept of *structured ambivalence* (13) highlights the internal conflict many women caregivers experience, where cultural expectations of care, combined with limited financial and social resources, can create conflicting pressures and emotional strain even when caregiving is deeply valued (14).

Despite well-established gender differences in caregiver burden, findings on gender and use of paid services are inconclusive. Vipperman et al. report no significant gender difference in paid service utilization among rural dementia caregivers (7). Others have found that differences depend on the type of service. For example, Sun et al. (15) found that men were more likely to use in-home care, while women were more likely to use transportation services; no differences were found in the use of day care and support groups. Qualitative studies offer more nuanced insights into how men and women engage with services (16). For example, Brown et al. (17) noted that husband caregivers often seek help earlier and adopt a managerial approach to caregiving, while in another paper (18), they noted that wife caregivers tend to minimize problems they experience and are more likely not to seek help because they believe they are not too difficult to handle. These patterns suggest that caregiving strain may prompt different help-seeking responses across genders.

The current study is guided by the Stress Process Model, which conceptualizes caregiving stress as arising from both primary demands (e.g., care tasks) and secondary strain (e.g., emotional distress) and emphasizes the role of coping resources, such as paid services, as potential buffers of stressors (19). Within this framework, gender is treated as a contextual factor that influences not only caregivers' exposure to stress but also their access to, and use of, paid support.

Building on this model, our study has two primary objectives:

1. To examine the association between spousal caregivers' experiences of loneliness and overload and their use of paid in-home care services.
2. To assess whether these associations differ by gender.

We hypothesize that the use of paid in-home services will be associated with lower levels of loneliness and overload, particularly among husband caregivers. We further anticipate that these associations will be weaker for wives, who may be experiencing greater internalized caregiving norms and therefore not seeking assistance. By addressing both emotional stress of caregiving and gendered service use patterns, this study aims to contribute to a more nuanced understanding of how to support the wellbeing of spousal caregivers in the context of dementia care.

## 2 Methods

### 2.1 Study design and participants

This study draws from a larger mixed-method, two-phase investigation (FACES-AD) that examined the caregiving experiences of family members providing care to PLwD in rural Appalachian counties of Virginia (20). A total of 539 screening calls yielded 233 eligible family caregivers, of whom 183 consented to participate. Twenty caregivers subsequently withdrew after consenting, primarily due to time constraints or acute health problems for the caregiver or PLwD, resulting in a final sample of 163 family caregivers. For the current analysis, we focused on Phase 1 structured telephone interview data of spousal caregivers.

Participants were included if they were (a) the spouse of a person diagnosed with dementia, (b) the primary caregiver involved in day-to-day care, and (c) residing in one of the 23 designated Appalachian counties in Virginia. Additional inclusion criteria included English fluency, telephone access, and a minimum of 10 years of residence in the region. Of the initial sample, 74 spouse caregivers participated (30 husbands, 41%; 44 wives, 59%), and after applying inclusion criteria specific to the current study's analysis, 61 caregivers (23 husbands, 38%; 38 wives, 62%) comprised the final analytic sample.

### 2.2 Recruitment and procedures

Caregivers were identified through clinical referrals from a large health care system and through local Area Agencies on Aging serving the targeted counties. Recruitment followed a two-step procedure. First, families were mailed study information and were given the option to decline participation. Subsequently, those who did not opt out were contacted by trained interviewers by telephone to confirm eligibility, obtain consent, and schedule the interview. Trained research assistants conducted structured telephone interviews. Each interview lasted ~60 min and asked questions about caregiving roles, service use, stress, and wellbeing. Data were collected between 2017 and 2019. Institutional Review Boards of [anonymous] Clinic (IRB #19-627) and [anonymous] (IRB#16-776) approved this study.

### 2.3 Sample characteristics

The sample of spousal caregivers (*M*_age_ = 72 years, SD = 9.39, Range = 43–89 years) was predominantly White (98%), consistent with regional demographics, and represented long-standing marriages (*M* = 43 years, SD = 17.32, Range = 6–69 years). The majority of caregivers had a high school diploma/GED or some college education (46%), with ~38% having an associate's, bachelor's or advanced degree. Most caregivers (57%) had annual household incomes under $40,000, and a majority (89%) were not actively employed, being either retired, homemakers, or on disability. Approximately one-third (28%) of participants reported having “just enough money, with none left over,” indicating financial strain. The caregiving duration ranged from 3 months to 15 years, with nearly two-thirds (61%) providing care for three or more years.

### 2.4 Measures

We used key constructs from the Stress Process Model, focusing on caregiving stressors and the use of in-home paid services. We explain these measures below.

#### 2.4.1 Caregiving stressors

Two indicators of secondary stressors, overload and loneliness, were assessed. Overload was measured using the average of three items capturing physical and emotional exhaustion and limitations on personal time, including statements such as “Felt exhausted when you go to bed at night,” “Felt that you had more things to do than you can handle,” and “Felt that you did not have time just for yourself.” Caregivers responded on a four-point Likert-type scale (1 = Never, 4 = Often), with higher scores indicating greater overload (α = 0.81) (19). Loneliness was assessed with two of the original three items from the validated three-item UCLA Loneliness scale (21)—“How often do you feel left out?” and “How often do you feel isolated from others?”—plus one item we slightly adjusted for our rural caregivers, “How often do you feel lonely?” We substituted this wording for the original “How often do you feel that you lack companionship” after pre-testing feedback showed that the latter phrasing was unclear to caregivers. Together, the three questions capture the social (left out, isolated) and emotional (lonely) facets of the loneliness definition, but not the existential facet. Caregivers rated these items on a four-point Likert-type scale (1 = Never, 4 = Often), with higher average scores indicating greater loneliness (α = 0.80). Caregivers were classified into ‘*high-stress'* and ‘*low-stress'* groups using median splits based on the sample distributions. Specifically, caregivers scoring at or above the median (50th percentile) were categorized as ‘*high-stress'* (overload: scores ≥2; loneliness: scores ≥1.667), whereas those below these medians were categorized as ‘*low-stress'* (overload: scores < 2; loneliness: scores < 1.667). The sample mean and standard deviation were 1.95 (SD = 0.88) for overload and 1.63 (SD = 0.92) for loneliness. Caregivers with scores above the median on only one of the variables were not included in the analyses (*n* = 13).

#### 2.4.2 In-home paid care

The primary outcome was the use of in-home paid care, defined as receipt of any formal assistance with activities of daily living (ADLs) provided at home. Caregivers reported whether they used specific services. The proportion of caregivers who reported using each service was as follows: 43% used respite care, 25% used homemaker assistance, 25% used personal care services, 15% used home health nursing, and 5% used meal delivery. A binary variable was created to indicate the use of in-home paid care, coded as 0 for caregivers who did not use any in-home paid services, and 1 for those who used at least one of these services.

#### 2.4.3 Covariates

Two covariates were included to account for contextual influences on caregiver stress and service use. The functional status of the PLwD was assessed using the caregiver's report of their limitations in ADLs, including self-care tasks such as bathing, dressing, eating, grooming, toileting, and transferring in and out of bed. Each activity was rated on a five-point scale (1 = does not need help, 2 = needs reminders or a little help, 3 = needs a lot of help, 4 = cannot do on their own, and 5 = never did or not applicable). This variable represents a primary stressor within the Stress Process Model, capturing the intensity of daily care demands. Responses coded as ‘5′ were treated as missing, and the remaining items were reverse-coded and summed, with lower scores reflecting poorer functioning and greater need for assistance (α = 0.93) (22).

Perceived informal support was assessed using eight items reflecting the caregiver's perception of support from family members (23). Items captured both positive (e.g., “How much can you rely on them to help if you have a serious problem?”) and negative (e.g., “How often do they let you down when you are counting on them?”) aspects of support. Caregivers responded on a four-point Likert-type scale (1 = Not at all, 4 = A lot), with higher scores indicating more perceived support, which represents an enabling resource that may reduce reliance on paid care (α = 0.80).

### 2.5 Analytic strategy

*T*-test statistics were used to examine gender differences in overload, loneliness, and use of in-home paid services. A subgroup analysis was also conducted to compare service utilization among spousal caregivers classified as high-stress vs. low-stress using Fisher's exact test. The Firth logistic regression model, which is a highly effective method for handling rare events and small sample sizes (24), was used to estimate the odds of using in-home paid services by husband and wife caregivers classified into low- and high-stress groups. Husband caregivers in the low-stress group served as the reference category. The model also included ADL limitations of the PLwD and caregivers' perceived support from family members as covariates. Variance inflation factors (range: 1.17–1.97, all < 10) indicated no multicollinearity. Parameter estimates, standard errors, and odds ratios (OR), and 95% confidence intervals (CI) are reported. All analyses were conducted using Stata 18.

## 3 Results

### 3.1 Gender differences in caregiving stress and in-home service use

**Figure 1:**
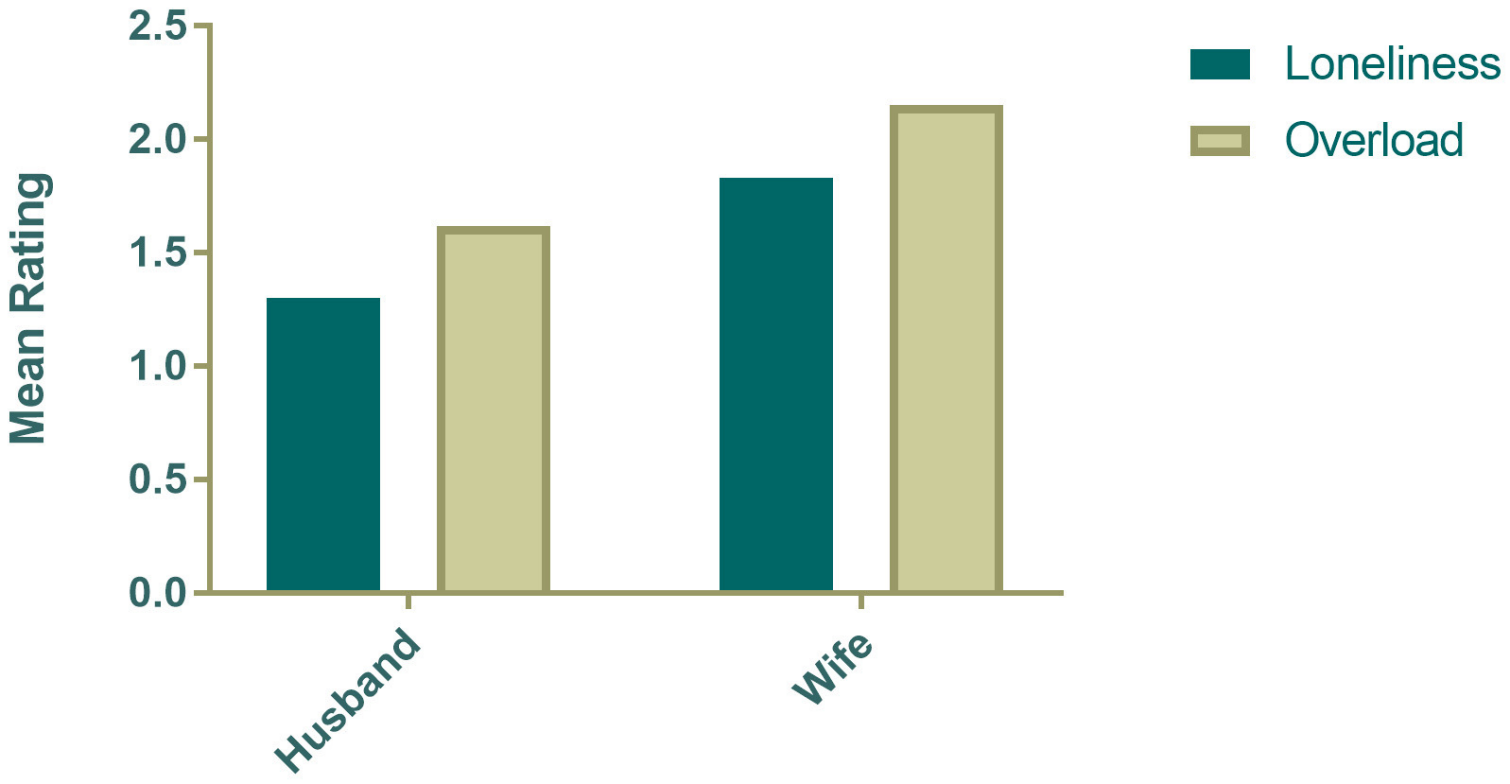
Gender differences in caregiver loneliness and overload among spousal dementia caregivers.

**Figure 2:**
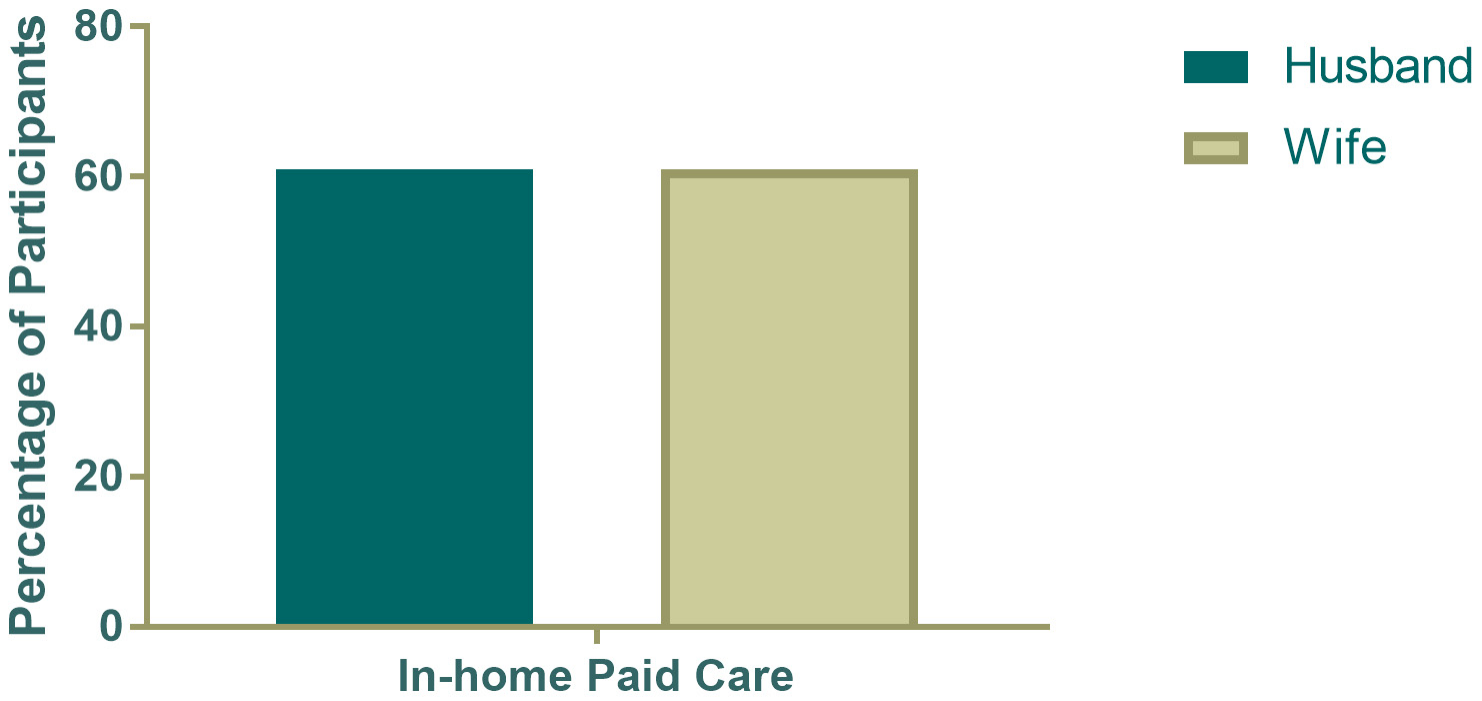
Use of paid in-home services by gender of spousal dementia caregivers.

As shown in Figures 1 and 2, husbands reported significantly lower levels of loneliness (*t* = −2.25, *p* = 0.03) and caregiver overload (*t* = −2.35, *p* = 0.02) than wives. However, in-home service use did not differ by gender: 61% of husbands and 61% of wives reported using paid in-home services.

### 3.2 Stress patterns and in-home service use

**Figure 3:**
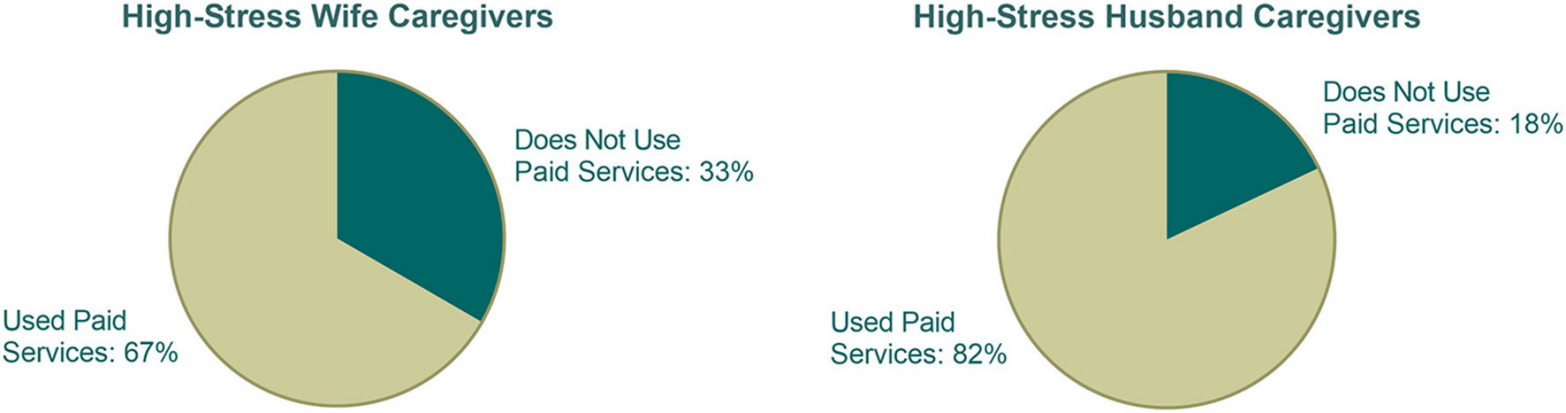
Use of paid in-home services by gender among high-stress caregivers.

Among the full sample of spouses, 35 caregivers (57%) were classified as experiencing high levels of both overload and loneliness. Among this high-stress group, 71.43% reported using in-home services (not shown), with 81.8% of high-stress husbands and 66.7% of high-stress wives reported using in-home care services (Figure 3). Although the proportion was higher among men, the difference was not statistically significant (Fisher's exact test, *p* = 0.45).

### 3.3 Logistic regression model

Results from the Firth logistic regression model are presented in Table 1. Compared to low-stress husbands, high-stress husbands were significantly more likely to use in-home services (OR = 14.33, *p* = 0.04). High-stress wives were also more likely to use in-home services (OR = 8.01, *p* = 0.03). Caregivers who perceived more support from family and friends were more likely to use in-home services (OR = 1.24, *p* = 0.03), suggesting that informal and formal resources may function synergistically. Functional limitations in activities of daily living were a significant predictor of in-home service use. Specifically, caregivers were more likely to use in-home support when the PLwD had greater functional impairments (OR = 0.82, *p* = 0.002). The wide confidence intervals for some interaction terms reflect the small sample size and rare event distribution.

**Table 1:** Firth logistic regression predicting use of in-home care services by spousal caregivers.

**Predictors**	***b* (S.E.)**	**OR**	**95% CI**	***p*-value**
Activities in daily living	−0.20 (0.06)	0.82	0.73 – 0.93	0.002
Family support	0.22 (0.10)	1.24	1.03 – 1.50	0.026
**Overload/loneliness by gender**				
Husbands, low overload/loneliness	Ref.	Ref.	Ref.	Ref.
Wives, low overload/loneliness	0.59 (0.93)	1.80	0.29–11.11	0.525
Husbands, high overload/loneliness	2.66 (1.33)	14.33	1.07–192.46	0.045
Wives, high overload/loneliness	2.08 (0.95)	8.01	1.26–51.10	0.028
Constant	−3.07 (2.71)	0.05	0.0002–9.38	0.257

b, Coefficient; S.E., Standard error; OR, Odds ratio; CI, Confidence interval; Ref., reference group. Model fit: Penalized log-likelihood = −22.33, Wald Chi^2^(5) = 13.30, *p* = 0.021. Wide confidence interval for interaction terms reflect small sample size and rare events.

## 4 Discussion

This study examined the associations among caregiver stress, gender, and the use of paid in-home care services among spousal caregivers of PLwD in rural Appalachia. Consistent with previous research (10, 11), wives reported significantly higher levels of caregiver overload and loneliness than husbands. The overall rates of in-home service use, however, were comparable for husbands and wives. More importantly, among caregivers classified as experiencing high emotional strain marked by elevated loneliness and overload, a higher percentage of husbands reported using paid in-home services compared to wives; however, the difference was not statistically significant, possibly due to small subgroup sample size. Nevertheless, these findings suggest gendered nuances in how emotional strain influences help-seeking behavior.

A key finding of this study was that both the subjective experience of caregiver stress (overload and loneliness) and objective caregiving demands (ADL limitations) were independently associated with in-home service use. Similar to previous research (25) and current eligibility criteria for paid care services in many states (26), caregivers in our study were more likely to use paid support when the PLwD had greater functional limitations. We also found that high levels of emotional strain, regardless of functional impairment, were strongly related to service utilization. This underscores the significance of secondary stressors such as caregiver loneliness and overload as meaningful indicators of caregivers' need for assistance from others.

The gender differences in service use, particularly within the high-stress subgroup, align with previous qualitative studies, suggesting that gender of the caregiver shapes service use (16–18). As suggested by these studies, men may be approaching caregiving tasks more pragmatically or managerially, viewing paid assistance as a logical resource to delegate tasks they feel ill-equipped or unwilling to handle. Women, on the other hand, may be internalizing cultural norms around caregiving, and therefore delaying the use of formal support despite experiencing high emotional strain. Although our study shows that many high-stress wives used in-home services, their usage was relatively lower to high-stress husbands, although not statistically significant, suggesting potential internal barriers, such as concerns about giving up caregiving responsibilities—albeit temporarily, discomfort with in-home workers, or guilt related to seeking external help. These results provide empirical support for Connidis and McMullin's *structured ambivalence* framework, highlighting that gendered caregiving expectations may heighten emotional stress, particularly among women caregivers (13, 14).

Our findings have implications for developing gender-sensitive interventions and policies. Current criteria for paid care services are primarily based on care recipients' functional impairments; our results argue for expanding these criteria to incorporate caregiver wellbeing, particularly emotional strain indicators such as loneliness and overload. Such a change could help better align service eligibility with caregivers' lived experiences and potentially mitigate caregiving-related emotional distress that could exacerbate poor health outcomes for the caregiver and unmet needs among PLwD. Additionally, community outreach and marketing efforts should acknowledge gender-specific service-use pathways. Educating men about the practical, task-oriented nature of the paid services might enhance timely uptake. For women, normalizing help-seeking, addressing guilt or stigma associated with asking for assistance, and ensuring services feel culturally acceptable, trustworthy, and aligned with personal caregiving standards may resonate more.

Several limitations warrant caution. We had a modest sample size recruited from a single geographic area, which limits the generalizability of our findings beyond rural Appalachia. Although our loneliness measure demonstrated good internal consistency, we did not use the full, unmodified three-item UCLA Loneliness Scale. Consequently, our scores may not be directly comparable with studies that use the unaltered instrument. Moreover, the cross-sectional design restricts conclusions about the directionality of observed associations between stress and in-home service use. Lastly, we conceptualized service use as a binary variable in this study. Future studies could incorporate more nuanced service utilization measures, such as service use intensity, attitude toward service use, and caregiver satisfaction with services, to get to a deeper understanding of service utilization among high-stress spousal caregivers.

In summary, this study underscores the complexity of spousal caregiving, highlighting the significant role of overload, loneliness, and gender in shaping caregivers' use of paid care services. Tailoring services and outreach to better address gendered caregiving norms and emotional strain can improve the accessibility and uptake of supportive care services by spousal caregivers in rural, under-resourced regions. Future research is essential to further disentangle these relationships and inform targeted interventions to enhance caregiver wellbeing and sustainability.

## Data Availability

The study data are not available because the primary investigators have not completed their original work with the dataset. Requests to access the datasets should be directed to JS, JSavla@vt.edu.
